# Editorial: Community series in insights into new strategies to combat biofilms, volume II

**DOI:** 10.3389/fmicb.2023.1198807

**Published:** 2023-06-27

**Authors:** Vishvanath Tiwari, Silvia Buroni, Luis Cláudio Nascimento da Silva, Sujogya Kumar Panda

**Affiliations:** ^1^Department of Biochemistry, Central University of Rajasthan, Ajmer, Rajasthan, India; ^2^Department of Biology and Biotechnology, University of Pavia, Pavia, Italy; ^3^Laboratory of Microbial Pathogenesis, Universidade Ceuma, São Luís, Brazil; ^4^Centre of Environment Climate Change and Public Health, Rashtriya Uchchatar Shiksha Abhiyan (RUSA) 2.0, Utkal University, Bhubaneswar, India

**Keywords:** biofilm, drug tolerance, combinational therapies, antifungal agents, antimicrobial agents

The emergence of antibiotic-resistant bacteria has become a major concern in healthcare and public health. It is particularly challenging to treat ESKAPE pathogens as they can form biofilms, which are about 1,000 times more resistant to antimicrobials as compared to planktonic cells. Therefore, alternative strategies are urgently needed to combat these pathogens (Roy et al., 2018; Tiwari, 2019; Panda et al., 2021). Biofilms form a complex layer with defined structures that attach on biotic or abiotic surfaces; they are particularly tough to eradicate and tend to cause resistance against most antibiotics (Sahoo et al., 2021). In general, biofilm-associated infections are a major public health concern, and the development of novel and effective strategies to combat them is essential. In the second edition of this Research Topic, five additional articles describing alternatives to treat biofilms have been published and are introduced here.

Boya et al. screened 83 indole derivatives to find compounds with antibiofilm activities against Uropathogenic *Escherichia coli* (UPEC). Among the screened compounds, chloroindoles-4-chloroindole (4CI), 5-chloroindole (5CI), and 5-chloro 2-methyl indole (5CMI) were indicated as the most active molecules as they showed minimum inhibitory concentrations (MICs) of 75 μg/mL, and inhibited more than 64% of UPEC biofilm formation at 20 μg/mL concentration. In addition to antibiofilm properties, the compounds showed activity against motility, curli formation, cell surface hydrophobicity (which favors bacterial adhesion to various surfaces), and indole production. Moreover, in the presence of indole compounds, the expression of other virulence genes, such as those involved in adhesion (e.g., *papA*), stress regulation (e.g., *csrA*), and iron uptake (e.g., *entE*), was downregulated. These findings render the molecules of great interest, especially for the treatment of polymicrobial biofilms.

In recently reviewed literature by Panda et al. the role of natural molecules such as antimicrobial peptides, bacteriophage endolysin, and essential oils against the biofilms formed by ESKAPE pathogens has also been discussed. The major focus of the review is on the anti-biofilm activity of the essential oils and their components. This review also critically discussed the other mode of actions i.e., disruption of biofilm and their inhibitory concentrations, expression of genes involved, other virulence factors etc. Tea oil, eugenol, citral, carvacrol, (+)-limonene were found to inhibit biofilm in methicillin resistant *Staphylococcus aureus* (MRSA). With transcriptome analysis, both tea oil and eugenol, confirmed the involvement of *sarA* gene (encodes the DNA-binding protein *SarA*), which is downregulated, and responsible for biofilm formation (Zhao et al., 2018) in addition to other genes e.g., enterotoxin gene (seA), and adhesion gene (icaD) (Yadav et al., 2015). Cinnamaldehyde is able to reduce biofilm in Gram-negative bacteria e.g., *Pseudomonas aeruginosa*, due to the probable reduction of N-acyl-homoserine lactone (AHLs) production (Chang et al., 2014), while was later confirmed that the inhibition is not due to its anti-quorum Sensing effect, but to its cytotoxic effects (Firmino et al., 2018).

Interestingly, various nanotechnology-based approaches have been developed to combat biofilms, including nanoparticles, nanofibers, and nanocoating (Al-Jamal and Kostarelos, 2011). Mohanta et al. discussed the potential of nanotechnology-based approaches to overcome antibiotic resistance and enhance the efficacy of conventional antimicrobial agents and the related challenges. Authors have addressed issues such as toxicity, stability, and biocompatibility of nanomaterials. They have also discussed potential solutions to these challenges, such as developing targeted nanomaterials and using appropriate quality control measures.

In a paper, Kaul et al. investigated the antibiofilm and antimicrobial properties of combinational therapy with Diethyldithiocarbamate (DDC) and Cu^2+^ complex against *Staphylococcus aureus* and *Staphylococcus epidermidis* biofilms. DDC is the metabolite of disulfiram and an FDA-approved drug for oral treatment of chronic alcoholism, which was previously investigated for its antifungal and anti-bacterial properties. Initially, the authors reported that anti-*S. epidermidis* action of DDC was substantially increased in the presence of Cu^2+^. Further, authors showed the combination of DDC and Cu^2+^ at different proportions could disturb the mature biofilms (24 h) formed by *S. epidermidis* or *S. aureus* strains. The combination also prevents bacterial attachment, biofilm growth under flow conditions and showed synergistic and additive effects with different classes of antibiotics. The combination was able to prolong the lifespan of *Galleria mellonella* larvae infected by *S. epidermidis* or MRSA strains. The authors' hypothesis for the antibacterial action of Cu (DDC)_2_ complex is the inhibition of the efflux transporter, one of the copper homeostasis components, leading to toxicity mediated by Cu^2+^ accumulation into bacteria. Furthermore, the excess Cu^2+^ could down-regulate the expression of *agr* and *sae* and other positive biofilm formation regulators.

Interestingly, in another study, Andriani et al. reported that BTU01 (a derivative of N-butylcarbamothioyl benzamide) exhibited antifungal activity with MIC (31.25–62.5 μg/mL) for planktonic cells, and 2 to 4-fold higher for sessile cells of *Cryptococcus neoformans* (125–1,000 μg/mL), being not toxic to mammalian cells. Due to its potent activity, as well as synergetic interaction with Amphotericin-B, authors followed up molecular docking studies and interesting results showed a strong interaction with enzyme-urease. Microscopic studies (Confocal laser scanning microscopy) also confirmed the reduction in the cell numbers and capsule size in planktonic state when treated alone or in combination with Amphotericin-B. All these *in vitro* results are interesting and further studies warrant an *in vivo* model with the mechanism of action to develop this thiourea derivative as a novel drug to control *C. neoformans* infections.

In conclusion, the emergence of antibiotic-resistant bacteria has led to the urgent need for alternative strategies specially to combat biofilms. Various approaches, such as essential oils, nanotechnology-based tools, and combinational therapy have shown promising in combating bacterial and fungal biofilms. The search for novel molecules and natural compounds that can lower virulence and reduce the expression of virulence genes continues. Further research is needed to optimize these approaches, enhance their efficacy and safety, and translate them into clinical practice. In the end, we would like to thank all the reviewers for their comments that improved our manuscripts, and the authors for their excellent contributions. We hope that this article Research Topic will inspire scientists from different fields of research focusing on biofilm.

## Author contributions

All authors listed have made a substantial, direct, and intellectual contribution to the work and approved it for publication.
